# Wireless Passive Ultra High Frequency RFID Antenna Sensor for Surface Crack Monitoring and Quantitative Analysis

**DOI:** 10.3390/s18072130

**Published:** 2018-07-03

**Authors:** Jun Zhang, Bei Huang, Gary Zhang, Gui Yun Tian

**Affiliations:** 1 School of Information Engineering, Guangdong University of Technology, Guangzhou 510006, China; garyzhang@gdut.edu.cn; 2 School of Materials and Energy, Guangdong University of Technology, Guangzhou 510006, China; 1111702006@mail2.gdut.edu.cn; 3 School of Engineering, Newcastle University, Newcastle upon Tyne NE1 7RU, UK; g.y.tian@ncl.ac.uk

**Keywords:** antenna sensor, circular patch, crack characterization, ultra-high frequency (UHF), radio frequency identification (RFID), structural health monitoring (SHM)

## Abstract

An exponential increase in large-scale infrastructure facilitates the development of wireless passive sensors for permanent installation and in-service health monitoring. Due to their wireless, passive and cost-effective characteristics, ultra-high frequency (UHF) radio frequency identification (RFID) tag antenna based sensors are receiving increasing attention for structural health monitoring (SHM). This paper uses a circular patch antenna sensor with an open rectangular window for crack monitoring. The sensing mechanism is quantitatively studied in conjunction with a mode analysis, which can uncover the intrinsic principle for turning an antenna into a crack sensor. The robustness of the feature is examined when the variation of crack position associated with an aluminum sample and the antenna sensor is considered. The experimental results demonstrate a reasonable sensitivity and resolution for crack characterization.

## 1. Introduction

Structural health monitoring (SHM) enabling with wireless sensor networks (WSNs) is widely used for condition-based maintenance of large-scale infrastructure [1]. Despite the fact that engineering components or structures are intensively designed against fatigue failures, more than 50% of mechanical failures are due to fatigue crack [2], which may cause the economic loss and jeopardize the human safety. Due to the development and maturity of internet of things (IoT), wireless passive sensors are highly desirable in large-scale infrastructure for ubiquitous and life-long monitoring [3].

Power consumption and power harvesting/scavenging capability are two issues in the design and development of wireless passive sensors. Recently, simultaneous wireless power and information transfer (SWIPT) is receiving increasing attention in fifth generation (5G) cellular network [4,5,6]. Because of continuous scaling down of power consumption of integrated circuits, backscattering communication using scavenging energy from 5G base station can make ubiquitous sensing possible. The concept of power harvesting and backscattering communication has been verified in the remote TV station assistance ambient backscattering [7,8,9].

Enabling with power harvesting from electromagnetic (EM) source, radio frequency identification (RFID) tag antenna based sensors are a promising candidate to be developed into wireless passive sensors [10,11,12]. Antenna sensors in ultra-high frequency (UHF) band provide benefits of ultra-low cost in crack monitoring, whose evolution can be found in [13]. In the absence of power consumption from sensing component, the communication range of this type of sensor can reach up to ten meters. Therefore, this type of sensor can be used to monitor crack growth in large-scale infrastructures instead of other traditional technologies with wire and battery.

Since the sensing information is extracted from EM signature of antenna sensors through backscattering communication in the UHF RFID band, the RFID sensing system is quite susceptible to interferences from wireless channel [14]. This scenario becomes worse for chipless RFID antenna sensors. Considering the quality of service (QoS) in the big data, the sensor design and interrogation method are critical for the source reliability of IoT [15].

Because the detuning based antenna sensor detects defect through varying the tag antenna’s impedance and gain, its sensitivity, resolution and robustness are influenced by the following factors. First, the defect detection underneath the antenna sensor is closely related to its mode and relative position between antenna and defect. This challenges the practical installation when the location of defect is unknown in priori. Second, the basic EM signatures suitable for sensing purpose are constrained. More to the point, the EM signatures, for example, power and phase, transfer the sensing information through backscattering in an analog form; hence, the sensing information is quite susceptible to interference and noise from both wireless channel and receiver. Third, the limited bandwidth in the UHF band imposes a tradeoff between sensitivity and dynamic range under a constant distance constraint. In addition, the multiple influences from both sensing and communication needs physical-based signal processing method for reliable feature extraction [16].

Benefiting from low profile and low cost, patch antennas were used for crack sensing [17]. The sensing principle for this type of antenna sensor is straightforward: a presence of crack in the ground plane along the width direction of patch antennas increases the current path in the length direction. It causes a resonant frequency shift to the lower frequency when compared with the original one without the presence of crack [18]. The work in crack monitoring based on antenna sensors falls into two categories: crack initiation detection and crack growth characterization [19].

The severity of the failure depends on both the crack length and orientation with respect to the loading direction. Longitudinal cracks are the most common and dangerous cracks because they can reduce a structure’s cross section and therefore reduce its structural capacity/integrity. A patch antenna sensor was proposed to monitor the growth of crack in a sub-mm resolution [20]. The crack orientation was detected using dual-mode operating patch [21]. Meanwhile, a multiplexing antenna sensor was designed to detect a multi-site crack [22]. Recently, a frequency-coded chipless antenna sensor with high-temperature-resistant capability was developed for crack orientation and width monitoring. Fatigue crack was also proved to be detectable in a sub-mm resolution [23]. Nevertheless, the above systems were not compatible with the EPC Global C1G2 standard. An experimental result demonstrated that the patch antenna sensor was capable of measuring sub-mm cracks and tracking crack propagation in the Federal Communications Commission (FCC) band [24].

In addition, the length and orientation of the crack can be detected using a 2D grid of conventional dipole tags in the UHF band with a commercialized RFID reader [25]. In spite of power, the backscattered phase can function as an EM signature. A sub-mm resolution was achieved in crack width detection using two coupled patch antennas [26]. However, the behavior of backscattered phase is dependent on the wireless channel, for example, propagation loss between reader and tag antennas, making it limited in the in-situ monitoring.

Above all, the feasibility of crack detection and characterization has been widely studied in the literature. However, the sensitivity and reliability of crack detection are tightly related to crack position with respect to antenna mode and the size of metal to be mounted. This paper aims to investigate the reliability of crack detection and characterization via the mode analysis of an antenna. The antenna sensor studied here is dedicated for monitoring the evolution of already existing cracks or of junctions prone to cracks [27]. The rest of this paper is organized as follows. First, the wireless interrogation and sensor setup are briefly described in Section 2. In Section 3, a mode analysis together with parametric study is investigated. The experiments are carried out and discussions are made in Section 4. The last section concludes the research findings.

## 2. Wireless Interrogation and Sensor Setup

In this section, we first describe an interrogation method based on a measurable EM signature, named threshold power to activate tag, in the downlink of the UHF RFID system. Then, one patch antenna with an open rectangular window is designed for sensitive crack sensing.

### 2.1. Wireless Interrogation

Under the hypothesis of line-of-sight (LoS) propagation between reader and tag antennas, the threshold power to activate the tag in reader antenna, $P_{R}$, can be expressed as

(1) $$\;P_{R}={\left(\frac{4\pi d}{\lambda_{0}}\right)}^{2}\frac{P_{th}}{G_{R}\left(\Theta ,\Phi \right)G_{T}\left(\theta ,\varphi \right)\left[\Psi \right]\tau \left[\Psi \right]\eta_{\rho }},$$

where d is the distance between the reader and tag antennas, $\lambda_{0}$ is the free-space wavelength, $P_{th}$ is the minimum threshold power to activate the tag chip, $G_{R}\left(\Theta ,\Phi \right)$ is the gain of the reader antenna, $G_{T}\left(\theta ,\varphi \right)\left[\Psi \right]$ is the gain of the tag antenna and $\eta_{\rho }$ is the polarization mismatch between the reader and tag antennas. The power transmission coefficient, τ[Ψ], which accounts for the impedance mismatch between the tag chip $\left(Z_{c}=R_{c}+jX_{c}\right)$ and the tag antenna $\left(Z_{a}\left[\Psi \right]=R_{a}\left[\Psi \right]+jX_{a}\left[\Psi \right]\right)$, is given by

(2) $$\;\tau \left[\Psi \right]=1-{\left|S_{11}\left[\Psi \right]\right|}^{2}=1-{\left|\frac{Z_{c}-Z_{a}^{*}\left[\Psi \right]}{Z_{c}+Z_{a}\left[\Psi \right]}\right|}^{2}=\frac{4R_{c}R_{a}\left[\Psi \right]}{{\left|Z_{c}+Z_{a}\left[\Psi \right]\right|}^{2}}.$$

where * means the conjugate value, Ψ represents the crack defect and $Z_{a}\left[\Psi \right]$ is dependent on the defect to be monitored.

### 2.2. Sensor Setup

From the antenna theory, the radiating patch and the ground plane can form a resonant cavity, so the sensitive part of patch antennas can easily cover its underneath area. Increasing crack depth can increase the effective electrical length of the antenna and therefore leads a shift of resonance to the lower frequency region. By this way, the patch antenna can be easily turned into a crack sensor. Due to the proximity coupling, the sensing performance of an antenna sensor, however, depends on the crack position with respect to the antenna mode.

In general, the crack position may be hard to be predicted. The variation of sensitivity with crack position will affect the reliability of crack characterization. Therefore, the linearity in the sensing area is of paramount importance for robust crack monitoring. In this paper, a circular patch is used as a radiator. A rectangular window is opened in the center of the patch to increase the sensitivity when crack grows as well as to improve the robustness with respect to crack position. Meanwhile, the antenna sensor is designed for conjugate impedance matching to the tag chip of NXP UCODE G2iM+, the input impedance and typical read sensitivity of which are 21.2 − j199.7 Ω and −17.6 dBm at 915 MHz, respectively. The geometry of the antenna sensor used in this paper is similar with [28]. To reduce the cost, the material of antenna substrate is Flame Retardant 4 (FR4), with a relative permittivity of 4.4, a loss tangent of 0.02 and a thickness of 2.0 mm. The conjugate impedance matching can be easily achieved by tuning the length and width of the rectangular window.

**Figure 1:**
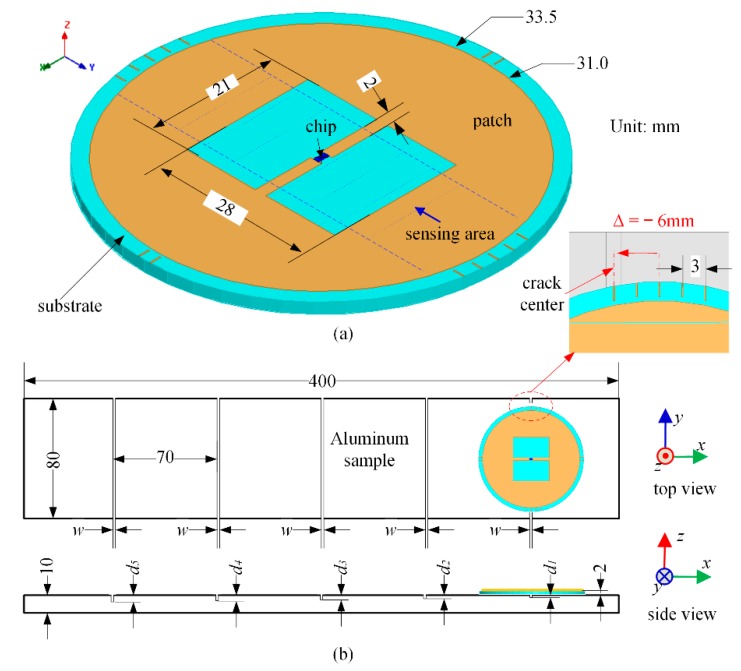
The antenna sensor and installation: (**a**) perspective view and (**b**) simulation setup.

The detailed size and simulation setup of the antenna sensor are shown in Figure 1. In order to evaluate the sensing performance, the depth of crack is progressively increased. The crack is modeled with a fixed width (*w*) of 2 mm and a variable depth of *d*. For reference convenience, the crack depth is represented *d*_1_ = 1 mm, *d*_2_ = 2 mm, *d*_3_ = 3 mm, *d*_4_ = 4 mm, *d*_5_ = 5 mm, respectively. The antenna sensor is directly put on the surface of the aluminum sample. The relative position between the antenna sensor and longitudinal crack moves from Δ = −6 mm to Δ = 0 mm for quantitative analysis of its robustness. Like any other sensor, the calibration is an important issue. Therefore, five positions in the backside of the aluminum sample corresponding to Δ = 0 were simulated to refer the healthy state (*d*_0_ = 0 mm) and also to study the influence of antenna performance with respect to the sample.

## 3. Mode Analysis and Parametric Studies

In this section, the current distribution is analyzed in conjunction with a parametric study to demonstrate a potential way leading to low-cost design of wireless passive sensors. The quality factor of the antenna is extracted and discussed to uncover the intrinsic sensing principle associated with further feature extraction.

### 3.1. Mode Analysis

**Figure 2:**
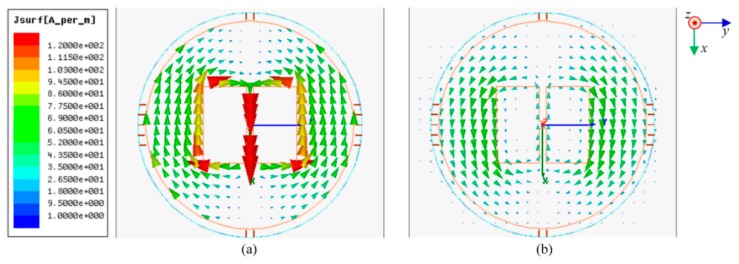
Simulated current distributions of the antenna sensor at 933 MHz on: (**a**) radiating patch and (**b**) metallic surface.

The simulated current distribution of the antenna sensor at the healthy state is displayed in Figure 2, where a shared scale is plotted on the left. The antenna sensor is excited with an identical 1-W input power at all frequencies. For an antenna operated at its fundamental mode, the current is maximized at the patch center and decreases along the patch length [29]. The radiation occurs at the two terminals of the patch. Therefore, it can be predicted that the sensing sensitivity is monotonically decreased from the center to radiating terminals.

**Figure 3:**
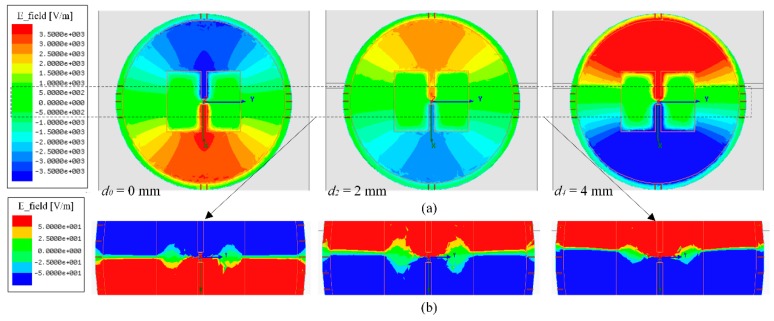
Simulated *z*-direction E-field of the antenna sensor at 920 MHz and Δ = −6 mm, where the field is extracted from the center of cavity formed by the circular patch and metallic surface: (**a**) full scale and (**b**) zoom in.

In general, a presence of crack will disturb the field distribution between the metallic surface and radiating patch. This perturbation will affect the radiation and stored energies, therefore, the resistant and reactant parts of input impedance. Figure 3 shows the simulated field distributions of the antenna sensor at a presence of metal with crack. We can find that a constant increase of crack depth will continuously shift the phase transition of the field distribution. This shift is contributed to the increase of the effective electrical length of the current flow on the metallic surface.

### 3.2. Parametric Studies

**Figure 4:**
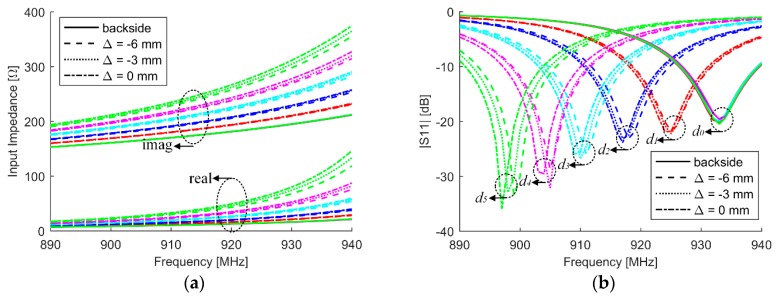
Simulated (**a**) input impedances and (**b**) reflection coefficients in the variations of crack depth and position, where solid line represents the backside reference when Δ = 0 mm.

Figure 4a,b provides the simulated input impedances and reflection coefficients of the antenna sensor in the variations of crack depth and position. The resonant frequency shifts to the lower frequency region when crack grows, which is agreed with conclusions from the mode analysis. The simulation results also indicate that the sensing sensitivity is maximized when the longitudinal crack is positioned at the center of the sensing area. It will slightly decrease as the crack moves away from its optimal position. Further simulation shows the sensitivity will decrease as crack orients toward the transverse. That is to say, the sensitivity of crack detection and characterization will decrease without in prior knowledge of the crack position and orientation. The worst case is that when the crack becomes transverse (*x*-direction) or moves outside of the patch, the crack cannot be detected.

On a lossy dielectric, the radiation efficiency of an antenna can be approximated using the antenna quality factor (*Q*) and the loss tangent of the dielectric, that is tanδ [30],

(3) $$\;\eta =\frac{R_{rad}}{R_{rad}+R_{loss}}\approx \frac{1}{1+Q\cdot \tan\delta }.$$

That is to say, the radiation efficiency might be improved by reducing the antenna *Q*. For a single resonance, the impedance of an antenna can imply its quality factor as [31]

(4) $$\;Q_{z}=\frac{\omega_{0}}{2R_{0}}\left|Z_{0}^{\prime }\left(\omega_{0}\right)\right|.$$

**Figure 5:**
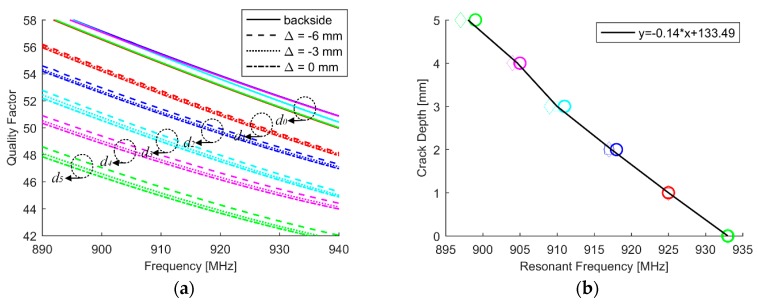
(**a**) Simulated quality factor in the variations of crack depth and position corresponding, where solid line represents the backside reference when Δ = 0 mm and (**b**) crack characterization.

For a conductor-backed dipole array, it was found that 58.8% and 62.5% of the total electric and magnetic energies were stored between the array and the ground plane, respectively [32]. The quality factor of the antenna at a presence of metal with defect is displayed in Figure 5a. We can find that the quality factor decreases with an increase of crack depth. And also, the quality factor is slightly dependent on the relative position between crack and patch. It can be predicted that the antenna gain of the antenna will be slightly improved as crack increases. The resonant frequency is extracted from Figure 4b and used as a feature to characterize the crack. The results are shown in Figure 5b. The first order curve fitting technique is utilized to extract the relation between the feature and crack, which can be used to quantify the crack depth once the resonant frequency of the antenna sensor is obtained. We can see that a 1-MHz decrease in the resonant frequency from the healthy state means a 0.14-mm increase in the crack depth.

**Figure 6:**
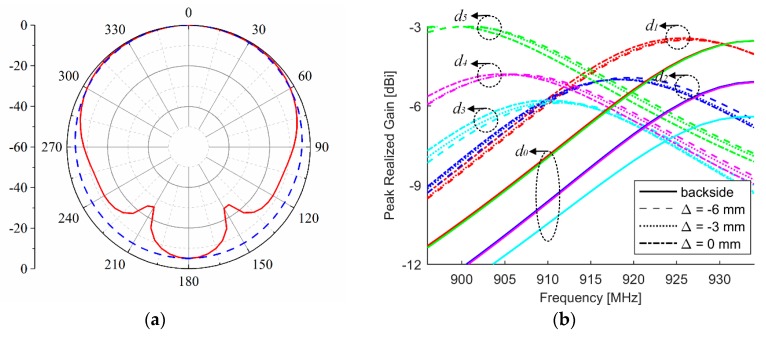
Simulated: (**a**) radiation pattern at the healthy state and 930 MHz, where solid line represents *xz*-plane and dash line represents *yz*-plane and (**b**) peak realized gain of the antenna versus frequency in the variations of crack depth and position, where solid line represents the backside reference when Δ = 0 mm.

The simulated radiation pattern and peak realized gain ($G_{T}\tau$) are depicted in Figure 6. As Figure 6a shows, the 3-dB beam-width in both *xz*- and *yz*-planes are larger than 120°. This broad beam-width makes the tag be easily identified. The resonant frequency of the antenna will monotonically decrease when crack grows. Meanwhile, the gain value will be continuously enhanced with the increase of crack depth, which is due to the constant improvement in the radiation efficiency. Nevertheless, we see from Figure 6b that the antenna position with respect to the small beam of the aluminum sample has significant influence on antenna’s radiation performance. At the healthy state, the antenna has the smallest gain at the center of the beam and the gain will increase when the antenna moves away from the center. That is to say, both crack position and depth will affect the maximum value of the antenna gain. However, the influence of crack position in the resonant frequency is not apparent. The peak realized gain of the antenna could reach its maximum value of 933 MHz at the healthy state. It reaches its maximum value of 898 MHz when *d*_5_ = 5 mm and Δ = 0 mm, which is a little higher than the value of *d*_1_ = 1 mm. This difference results from the depth increase. Therefore, the maximum sensitivity (at Δ = 0 mm) of the antenna sensor is 7.0 MHz/mm.

## 4. Experimental Studies and Results

### 4.1. Test Setup

**Figure 7:**
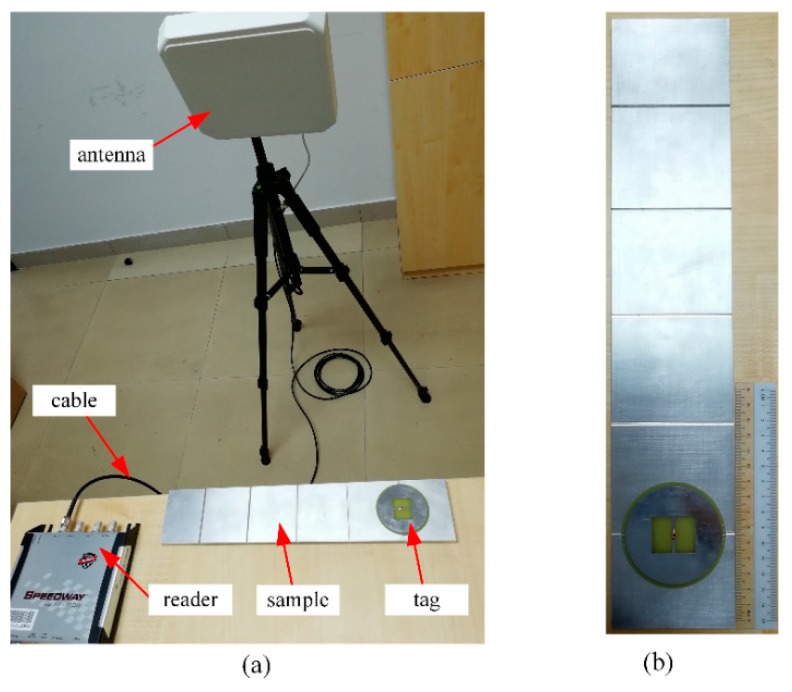
(**a**) Test setup of the UHF RFID sensing system and (**b**) schematic of the aluminum sample.

The test setup of the UHF RFID sensing system is depicted in Figure 7a. The measurement was carried out using a commercial RFID reader with an adjustable transmitting power. The reader antenna was circularly polarized (CP) with a 9 dBic antenna gain. The transmitting power input to the terminal of reader antenna was increased from 10 dBm in a step of 0.25 dB to find the threshold power. The maximum allowed input power was limited to be 27 dBm, yielding a maximum 4-W effective isotropic radiated power (EIRP). The measurement was carried out under the frequency range of 902.75–927.25 MHz with a resolution of 0.5 MHz. The communication distance between the reader and tag antennas was fixed at 1 m while the orientation of reader and tag antennas was kept fixed through all tests.

The schematic of an aluminum alloy 6061 sample sized of 400 mm × 80 mm × 10 mm is shown in Figure 7b. The detailed size of the beam is the same with Figure 1b. Five cracks were artificially made with an increasing crack depth from 1 to 5 mm in a step of 1 mm with a fixed crack width of 2 mm. The prototype antenna sensor was directly placed on the surface of the aluminum sample.

### 4.2. Results and Discussion

**Figure 8:**
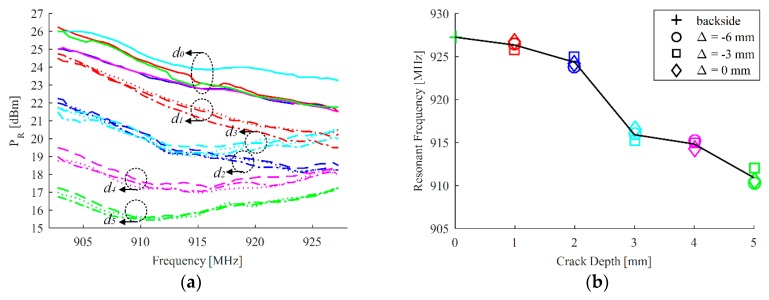
The measured threshold power to activate the tag in the variations of crack depth and position corresponding to Figure 6b: (**a**) where solid line represents the backside reference when Δ = 0 mm and dash line represents Δ = −6 mm, dot line represents Δ = −3 mm, dot dash line represents Δ = 0 mm with crack and (**b**) the corresponding features.

The defect influences the impedance of the antenna sensor, thus power transmission coefficient and therefore the threshold power to activate the tag varies with crack growth. Figure 8 plots the measured EM signature of $P_{R}$ versus frequency with different crack depths and positions and corresponding features of the resonant frequency. A 5-point moving average is utilized to smooth the data and therefore enhance the signal to noise ratio of $P_{R}$ for robust feature extraction. The resultant curves are displayed in Figure 8a. It could be noticeably observed that the resonance of the antenna sensor continuously shifts from the higher frequency region to the lower frequency region when crack grows. Therefore, there is a tradeoff between sensing and communication for wireless passive antenna sensors in the limited band. The curve shape of $P_{R}$ can partly reflect the reduction in quality factor as crack grows, which is agreed with the simulation. Meanwhile, we can find that the relative position of crack and sample has some not negligible effects on the quality factor of the antenna sensor, which is due to the influence from limited size of the ground plane. The variation in the minimum value of $P_{R}$ can also be observed when antenna moves from one beam edge to another. However, this relative position has a little influence on the resonant frequency of the antenna sensor.

For an in-situ monitoring application, the feature of $P_{R}$ in a fixed frequency point away from the resonance can be used to characterize the crack depth. However, this feature might be influenced by the beam size. At a sacrifice of sensitivity, the feature of the resonant frequency is more robust compared with feature of $P_{R}$ at a fixed frequency. The resonant frequency where the minimum threshold happens is extracted and displayed in Figure 8b. We can find that the sensitivity has a negligible variation when crack moves away from its optimal position in the range of sensing area. Due to the parasitic capacitance in the soldering between tag chip and tag antenna, the impedance mismatch occurs in the fabricated prototype, which causes a shift of resonant frequency at the healthy state and therefore leads to a lower sensitivity in the measurement when comparing with the simulated one. Meanwhile, the nonlinearity in the measurement can be observed, which is partly attributed to the resolution in the transmitter and also the interference from the wireless channel.

Considering the variations in geometry size and properties (e.g., permittivity and permeability), the fabrication error of the antenna and tolerance in the tag chip, the resonant frequency of the antenna sensor may shift [33]. In addition, the air gap between the metal and antenna due to the surface roughness or installation error, for example, glue used to firmly connect the antenna and metal, has a significant influence on the resonance. That is to say, we need to calibrate each sensor after its installation for robust and reliable crack monitoring. This calibrated data can be recorded thereafter to refer the healthy state.

## 5. Conclusions

The sensing performance of RFID tag antenna based sensor will degrade when the crack is not at its optimal position. In this paper, a circular patch antenna sensor with a rectangular window has been utilized for metal-mountable applications. The variation in the crack position is examined for quantitative investigation of robust crack monitoring. In conjunction with mode analysis, we find that the crack growth will constantly reduce the quality factor and therefore improve the realized gain of the antenna, where the profile is a key factor to determine the tradeoff between sensing and communication. Meanwhile, the size of the metallic geometry that the antenna sensors are going to be mounted has significant influence on their radiation performance, for example, directivity and efficiency. The experimental results validate that the uncovered sensing mechanism can provide potential guidance for robust feature extraction in longitudinal crack monitoring.

In this paper, the antenna sensor has been demonstrated to work on a small beam with longitudinal crack. However, the antenna sensor needs to be miniaturized to be mounted on a smaller beam; the beam may also become one piece of large-scale infrastructure. Without a reference tag, the antenna itself can be calibrated after the installation. Using the feature of resonant frequency, the monitoring may be taken automatically with unmanned vehicles in harsh environments.
